# An Energy-Efficient Approach to Enhance Virtual Sensors Provisioning in Sensor Clouds Environments

**DOI:** 10.3390/s18030689

**Published:** 2018-02-26

**Authors:** Marcus Vinícius de S. Lemos, Raimir Holanda Filho, Ricardo de Andrade L. Rabêlo, Carlos Giovanni N. de Carvalho, Douglas Lopes de S. Mendes, Valney da Gama Costa

**Affiliations:** 1 Computer Science Department, State University of Piaui, Rua Joao Cabral, 2231-Piraja, 64002-150 Teresina, Piaui, Brazil; cgnc@ctu.uespi.br (C.G.N.d.C.); valney.gama@gmail.com (V.d.G.C.); 2 Graduate Program in Applied Informatics (PPGIA), University of Fortaleza, Av. Washington Soares, 1321-Edson Queiroz, 60811-905 Fortaleza, Ceará, Brazil; raimir@unifor.br; 3 Graduate Program in Compupter Science (PPGCC), Federal University of Piaui, Ministro Petronio Portela Campus, 64049-550 Teresina, Piaui, Brazil; ricardoalr@ufpi.edu.br (R.d.A.L.R.); douglas_min@hotmail.com (D.L.d.S.M.)

**Keywords:** ant colony optimization, clustering, virtualization, wireless sensor networks

## Abstract

Virtual sensors provisioning is a central issue for sensors cloud middleware since it is responsible for selecting physical nodes, usually from Wireless Sensor Networks (WSN) of different owners, to handle user’s queries or applications. Recent works perform provisioning by clustering sensor nodes based on the correlation measurements and then selecting as few nodes as possible to preserve WSN energy. However, such works consider only homogeneous nodes (same set of sensors). Therefore, those works are not entirely appropriate for sensor clouds, which in most cases comprises heterogeneous sensor nodes. In this paper, we propose ACxSIMv2, an approach to enhance the provisioning task by considering heterogeneous environments. Two main algorithms form ACxSIMv2. The first one, ACASIMv1, creates multi-dimensional clusters of sensor nodes, taking into account the measurements correlations instead of the physical distance between nodes like most works on literature. Then, the second algorithm, ACOSIMv2, based on an Ant Colony Optimization system, selects an optimal set of sensors nodes from to respond user’s queries while attending all parameters and preserving the overall energy consumption. Results from initial experiments show that the approach reduces significantly the sensor cloud energy consumption compared to traditional works, providing a solution to be considered in sensor cloud scenarios.

## 1. Introduction

A Wireless Sensor Network (WSN) can be described as a set of small electronic devices (called sensor nodes) capable of sensing environment conditions (such as temperature, humidity, and light), processing and transmitting data wirelessly through a transceiver unit. These devices can also actuate on the environment, according to its programming. WSN has successfully been applied in several areas, such as healthcare [1,2], military [3], infra-structure surveillance [4,5,6], and environmental monitoring [7,8,9,10]. However, WSN have several constraints, mainly due to the nodes limitations [11]. In most cases, the sensors nodes have limited processing capability, memory, and bandwidth, and usually are battery powered [12]. These constraints hinder the development of WSN applications [13]. For example, WSN generally perform a specific task and the data monitored cannot be shared between users from different WSN. As a result, in recent years, researchers are taking advantages of cloud computing [14] to leverage the full potential of the WSN. The joint of these two technologies have given birth to the sensor cloud field.

**Figure 1:**
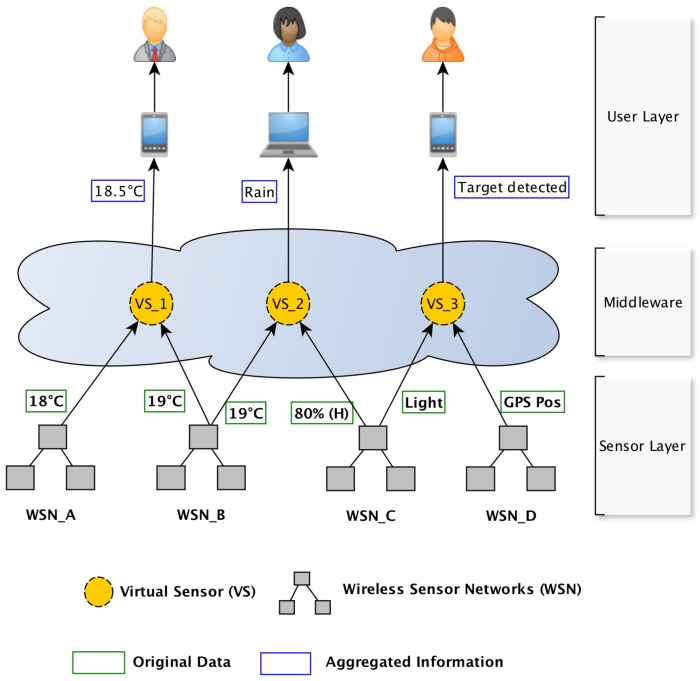
Conceptual model of a sensor cloud.

According to Madria et al. [15], a sensor cloud can be defined as a computing environment comprised of several wireless sensor networks (WSN) from different providers (owners), which may be distributed over a large geographical area. The cloud acts decoupling the physical sensors nodes from the users interested in the monitored data by managing virtual sensors. A virtual sensor is an emulation of a sensor that obtains its measurements from the physical sensor nodes present in the sensor cloud environment. Hence, multiple WSN can interoperate in order to attend single or multiple applications, and sensing activities can be provided as services for users, allowing them to acquire/purchase services on demand [16]. For example, a user interested in temperature and humidity measurements from a specific geographic location can request an instance of a virtual sensor in the sensor cloud and deactivate that instance when it is no longer needed. Thus, the user pays only for the resources they use. Figure 1 shows a typical sensor cloud infrastructure formed by three layers: the user-layer, middleware, and sensor-layer [15]. The user-layer serves as a gateway point for users while the middleware is responsible for managing the interactions between these users and virtual sensors. In Figure 1, three users interact with the sensor cloud by their instance of a virtual sensor. The sensor-layer is responsible for the registration and maintenance of sensor networks and the collection of data/measurements transmitted by the sensor nodes of these networks. Each WSN has an owner (organizations and people) and when a new owner decides to join the cloud, he must first publish his sensors nodes via some kind of metadata (for instance SensorML (http://www.opengeospatial.org/standards/sensorml)). In the same sense, the owner can stop the publishing when he does not want to share the sensor nodes anymore. The middleware layer is usually represented by an application server that receives all user queries (from the user-layer). The middleware reviews the user-defined parameters (such as sensing frequency, region of interest) in the query and checks for physical sensors (present in the sensor layer) capable of meeting these parameters. In the case of success, the middleware must decide, by a process called provisioning [15], which physical sensors should be allocated to form the virtual sensor. The virtual sensor is then bound to the query. If no sensors are available to process the query, the user should be informed. In this way, the user can try a new query based on different parameters.

Over the years, several proposals for specific middleware for sensor clouds environments have been published. Initial works [15,17,18] considered the provisioning of all nodes within the region of interest to send their measurements periodically to the middleware. However, such approach can lead to an overhead of messages, increasing the sensor node energy consumption (which is generally battery-powered). Nevertheless, recent works [19,20,21,22] proposed a different approach, clustering sensor nodes based on the similarity/correlation of the sensor nodes’ measurements, instead of physical distance, as many traditional works on WSN field [5,23]. Thus, the middleware can select only a small subset of nodes, whose measurements represent the most variance of the data, to start transmitting. However, such works have considered only homogeneous nodes, i.e., all nodes using the same set of sensors, making them not entirely suitable for sensor clouds environments.

**Figure 2:**
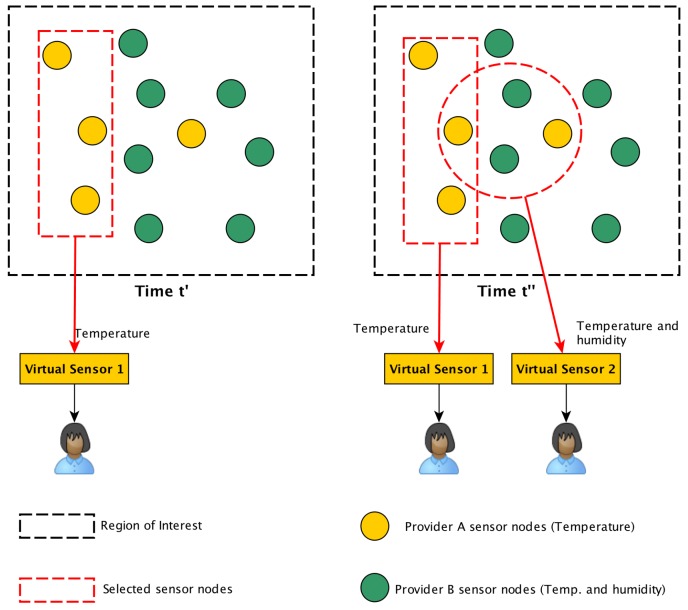
An example of provisioning

Because a sensor cloud connects several heterogeneous sensor nodes from different providers, the provisioning can be a complex task. As an example, consider Figure 2, which shows a sensor cloud environment composed of two WSN from different providers. The yellow nodes represent the *A* provider, while the green ones represent the *B* provider. The nodes from the *A* provider only have temperature sensors, while the nodes from the *B* provider have temperature and humidity sensors. First, at time $t^{\prime }$, a user requires temperature measurements from a specific region of interest (black lines in the figure). Then, at time $t^{\prime \prime }$, another user requires temperature and humidity measurements from the same region of interest. Consequently, the middleware must analyze and select from among all nodes the ones that are most suitable in order to create two virtual sensors, resulting in a combinatorial problem. Besides that, an algorithm for provisioning virtual sensors should be protocol independent since different WSN could use different technologies. For instance, a WSN could be based on IEEE 802.15.4 and Zigbee (http://www.zigbee.org) protocols layers while another WSN could be based on RF 433hz and some open routing protocol.

The objective of this paper is to present ACxSIMv2, an enhancement of our previous works [20,21] to fully supports the provisioning of virtual sensors in heterogeneous environments. ACxSIMv2 comprises two algorithms: (i) ACASIMv2 which clusters heterogenous sensor nodes by the correlations of their measurements; and (ii) ACOSIMv2, an algorithm based on Ant Colony Optimization [24] to select the optimal set of sensor nodes which will compose the virtual sensors required by users. ACxSIMv2 is independent of any routing protocol or technologies used by the WSN, and the optimal set of selected nodes has the following properties: (i) meet the queries requirements; and (ii) minimize the energy consumption of nodes that comprise the sensor cloud. The main contributions of this paper are:

1. The formulation of the provisioning task as an optimization problem. The defined model supports heterogeneous WSN and multiple queries/applications requested by users;
2. An algorithm to cluster heterogenous sensor nodes based on data similarity;
3. The assignment of the minimum set of sensor nodes that are compatible with the requirements of the user’s queries, and, at the same time, minimize the energy consumption; and
4. Through a comprehensive experimental study, it is shown that the proposed system achieves a significant improvement regarding the energy consumption of sensor nodes compared to the state-of-the-art approaches.

The article is structured as follows: Section 2 reviews some related works. Section 3 defines the system model and the problem definition. Section 4 presents the two algorithms that compound ACxSIMv2. Section 5 presents the methodology used to evaluate the performance of ACxSIMv2 and discusses the results. Finally, in Section 7, we present conclusions with some future research directions.

## 2. Related Works

The problem of virtual sensor provisioning appears similar to the sensor allocation problem [5,23,25,26,27,28,29,30], which is traditionally treated in the wireless sensor network context. However, these works consider only homogeneous sensor nodes (with the same physical characteristics such as the number and type of sensors) and a single application running on these nodes (for example, an application that periodically sends its measurements to the sink node). Another characteristic is the use of redundant sensors within the region of interest in order to organize the sensors into clusters. In the LEACH protocol [30], for instance, sensor nodes periodically change their status between sleep/wake-up. However, such a technique is not feasible in the sensor cloud environment since it does not consider different queries at the same time (for example, a case where query 1 requires temperature and humidity, and query 2 requires temperature and light) or sensor node heterogeneity (some nodes provide three types of sensors while others provide just one, for instance). Thus, there is no guarantee that awakened nodes can provide the required sensors. In Cloud Computing literature, there are already works on resource allocation, such as [31,32], in which they also consider the optimal selection of resources in datacenters (like virtual machines, storage disks, and memories) to save energy consumption. Although similar to virtual sensor provisioning, the algorithms found in cloud computing context cannot be used in sensor cloud, since they do not take into account the constrained and specifics characteristics of a WSN (such as the small processing capability and limited energy supply).

**Figure 3:**
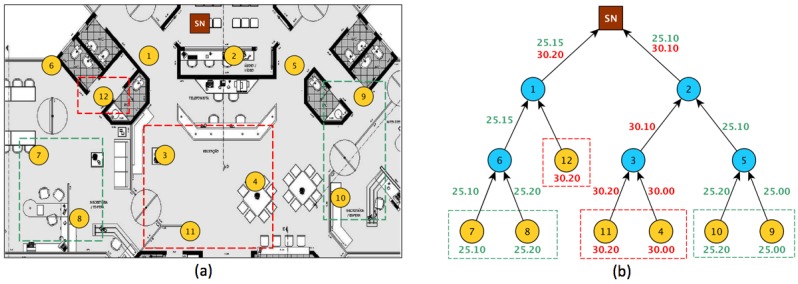
(**a**) Eleven sensor nodes deployed over a laboratory. (**b**) The 11 sensors build a routing tree. Notice correlated sensors nodes that are not close to each other.

As many applications are related to the periodic monitoring of physical conditions (such as temperature and humidity), redundant data may be sent by many sensor nodes [33]. Most works in the literature consider data aggregation to overcome this situation [8,34,35,36,37,38]. In these works, aggregation occurs when nodes are close to each other, or when data are relayed by some intermediate node, in some tree-routing scheme. However, the fact that nodes are close to each other does not guarantee data correlation in order to perform aggregation. For instance, two nodes can be separated by a wall. One node can be inside a room cooled by some air conditioning system, and the other one can be outside during a hot day. To illustrate that problem, consider the WSN in Figure 3a. There are 11 sensor nodes, represented by yellow circles, and one sink node, represented by a small brown square, at the top of the figure. The sensor nodes inside dashed squares are the selected ones to sensor the environment and to send their measurements. The others nodes are selected to act as a relay to the sink node. The color of the dashes represents regions whose temperature are very close, according to the user configuration. In Figure 3b, the nodes are organized in clustering-based routing scheme and the measurements transmitted by the sensor nodes are summarized by the relay-nodes. Although Nodes 7 and 8 are measuring temperature values very close to the values of the Nodes 10 and 9, Clusters 6 and 5 are transmitting the same values, generating an overhead of messages. In ACxSIM, that problem could be mitigated by the selection of only a subset of sensor nodes from “red” and “green” clusters. For example, if only Node 7 and Node 12 were selected to transmit their measurements, the user would still have a view of all the region monitored.

Chatterjee et al. [39] propose a scheme for the composition of virtual sensors based on the selection of only a subset of physical sensor nodes. The selection process takes into account the *goodness* of the physical sensor nodes, i.e., how good is a particular sensor node to be part of a solution. However, the proposed scheme tries only to minimize the number of sensor nodes capable of fulfilling the requirements of the applications, not considering the similarity and correlations between the nodes measurements. In this way, there is no guarantee that redundant nodes will not be chosen. In addition, inside a specific region, all sensor nodes must be homogenous. Hence, the virtual sensors comprise of homogeneous sensor nodes (although different virtual sensors, from different regions, may form a Virtual Sensor Group). In our solution, we do not impose these restrictions, since every WSN can be composed of heterogeneous sensor nodes and the measurements from the selected nodes can represent multiple geographical areas, based on the correlations between their measurements. In this way, the subset of nodes selected by the middleware will not contain redundant nodes, improving the network lifetime.

The authors in [40,41,42] introduce an interactive model for the sensor cloud environments to efficiently provide on-demand sensing services for multiple applications with different requirements at the same time. It is a novel concept since the middleware can now perform the aggregation of application requests to minimize the resource consumptions of the constrained physical sensor nodes while attending all the requirements of the users’ applications. Hence, with few queries sent to the sensor nodes, the middleware could ensure the responses needed by many applications. However, all nodes still receive the consolidated request and adapt their sensing interval in order to attend all applications. In our work, the middleware selects only a subset of physical sensor nodes to performing the transmissions of their responses. Nevertheless, we will investigate how to integrate ACxSIMv2 with the interactive model in future works.

Sarkar et al. [19] present a framework (Virtual Sensing Framework (VSF)) to predicts multiple consecutive sensor data while some the sensors are inactive, thus, reducing sensing and data transmissions activities. Similar to our work, VSF exploits the temporal and spatial correlations amongst sensed data to select the sensor nodes to respond the users’ queries. However, the work focuses only on WSN environments, not considering how the correlations could be done in sensor cloud environments with multiples WSN.

Dinh and Kim [43] consider the provisioning of virtual sensors by clustering sensor nodes not physically close to each other. Nevertheless, each node is responsible for just one type of sensor (for example, temperature or humidity, but not both), and the node selection is based on the nodes with the highest energy value. In ACxSIMv2, however, each sensor node can measure multiples variables and the selection process does not take into account only the energy parameter since, in a heterogeneous environment, nodes with higher energy can not necessarily attend all applications requirements.

The work in [22] explores the concept of multidimensional behavioral clustering [44] for reducing message transmission in traditional WSN. In that sense, the sink node creates virtual clusters by clustering the sensors nodes according to the correlations of the measurements from all monitored (dimensions). However, the work is not suitable for sensor cloud environments since it takes into account all dimensions at once to perform the clustering of nodes. In addition, the work uses the energy as a parameter to choose the nodes from the clusters. However, in a sensor cloud comprised of heterogeneous sensor nodes, there is no guarantee that sensor nodes with high energy could attend the user’s queries.

From the above-mentioned works, it is clear the lack of an efficient approach to support virtual sensors in sensor clouds. The traditional solutions for resources allocation in Cloud Computing and WSN context are not suitable, since they do not consider the specificities of sensor clouds. First works designed specifically for sensor clouds considered the provisioning of all sensors node, which is energy inefficient since redundant nodes can be selected. Recent works tried to exploit clustering but considering only homogeneous sensor nodes. Therefore, they are not appropriate for sensor cloud environments, which generally comprises nodes with different set of sensors devices. In ACxSIMv2, we consider the exploiting of temporal and spatial correlations between sensor nodes makes to create clusters based on the similarity of its measurements, but taking into account the sensor cloud heterogeneity. Consequently, there is a reduction in the selection of redundant nodes since one node can represent different areas from which it is deployed (Spatial correlation). Because of the temporal correlation between the measurements, the transmission of the selected nodes can be reduced since the middleware of the sensor cloud can predict (with high accuracy) consecutive sensing measurements. In this way, the overall energy consumption of the sensor nodes is reduced, prolonging the lifetime of the sensor cloud.

## 3. Problem Definition

In this section, we first describe some basic assumptions considered in this work, followed by the mathematical formulation of the problem. Table 1 shows the notations and their descriptions used in the rest of the paper.

### 3.1. Assumptions

This work focus on monitoring-type applications, in which all sensor nodes periodically gather information by monitoring an geographic area, denoted region of interest. The nodes can be heterogeneous, i.e., the nodes do not need to have the same set of sensors. The nodes also cannot change its positions after deployment (static nodes) [45]. It is considered that the provisioning task is performed by the middleware of the sensor cloud. The middleware has a local database that stores information about all the sensor nodes in the Sensor Cloud. This local database is updated whenever a new WSN is deployed in the Cloud or when a sensor node is disabled or added to an existing WSN. The middleware does not need to be aware of the routing protocol used by each WSN since only the sink node (base station) of each WSN has the responsibility of delivering the messages required to the middleware. Thus, whenever the middleware needs to create a virtual sensor, it must inform the selected sensor nodes, using the sinks as a proxy for the WSN. Once the nodes are selected, they start sending their measurements according to the received parameters.

### 3.2. The Problem Definition

A sensor cloud (SC) is a set of Wireless Sensor Networks (WSN) and can be defined as $SC=\{WSN_{1},WSN_{2},\cdots ,WSN_{N}\}$. Each $WSN_{i}$ is comprised of a set of sensors nodes $WSN_{i}=\left\{SN_{i,1},SN_{i,2},\cdots ,SN_{i,N^{i}}\right\}$, where 1≤i≤N and $N^{i}$ is the number of SN in the $WSN_{i}$, deployed in an area $T_{i}=H_{i}\times W_{i}$, where *H* and *W* are the height and width respectively. Each sensor node comprises a set of sensors devices denoted by $SN_{i,j}=\left\{S_{1}^{i,j},S_{2}^{i,j},\cdots ,S_{D^{i,j}}^{i,j}\right\}$, where $1\leq j\leq N^{i}$. A sensor device means the analog or digital circuit used to capture some physical or environmental conditions (temperature, light, etc.). Each sensor device $S_{k}^{i,j}$, where $1\leq k\leq D^{i,j}$, has a type/dimension $T_{k}^{i,j}=Type_{t}$, which denotes the physical condition it can sensors. The set of all dimensions monitored by the SC is $TYPE=\{Type_{1},Type_{2},\cdots ,Type_{N_{T}}\}$ ($1\leq t\leq N_{T}$). Given a sensor device, there is a set of measurements associated with it denoted by ($S_{k}^{i,j}=\left\{M_{1},M_{2},\cdots ,M_{M^{k,i,j}}\right\}$). The user’s query is defined by $Query_{id}=\left\{T,user,sample\_interval,time,R\right\}$, where id means the query identification, T⊂TYPE means the user’s interested dimension, user denotes the user identification, sample_interval denotes how often data are physically sampled, time represents how long the node has to sample the measurements, and *R* stands for *Region of Interest*, i.e., the geographical area in which the sensors nodes should act. Several queries can be requested. The set of all queries is $QUERY=\{Query_{1},Query_{2},\cdots ,Query_{N_{Q}}\}$, where $N_{Q}>0$.

**Definition** **1.** *Sensors ratio ($\kappa_{j,i,id}$). It is defined by the ratio between the number of the type of sensors requested by the user’s query ($Query_{id}$) and the number of the type of sensors provided by a particular sensor Node $SN_{j,i}$. Values close to 0 mean that more of the type of sensors needed is provided by a specific sensor node. SR is expressed as:*

(1) $$\kappa_{j,i,id}=\frac{\Phi (SN_{i,j})}{\Phi (Query_{id})}$$

*where* Φ(⋯) *defines a function to count the number of unique sensor types presents in a set.*

**Definition** **2.** *Normalized residual energy ($\lambda_{j,i}$). It is defined as the ratio of the current energy level to the initial energy level, expressed as,*

(2) $$\lambda_{j,i}={\left(\frac{E_{SN_{j,i}}^{cur}}{E_{SN_{j,i}}^{ini}}\right)}^{-1}$$

*where* $E_{SN_{j,i}}^{cur}$*, and* $E_{SN_{j,i}}^{ini}$ *are the current and the initial battery level, respectively.*

**Definition** **3.** *Proximity with BS ($\chi_{i,j}$). It measures how close a sensor Node $SN_{i,j}$ is to its base station $BS_{i}$ (based on the euclidian distance). It can be expressed as follows:*

(3) $$\chi_{j,i}=euclidian\left(SN_{i,j}.pos,BS_{i}.pos\right)$$

*where* <Node>.pos *computes the position (in terms of latitude and longitude) of the sensor node.*

**Definition** **4.** *Used Networks Ratio (η). This ratio takes into account the number of WSN used in a solution over the total number of WSN in the sensor cloud environment. The Used Network Ratio tries to avoid the overusing of some WSN regarding the others and can be defined as:*

(4) $$\eta =\frac{total\_of\_networks}{selected\_networks}$$

**Definition** **5.** *Selected Sensors Nodes (*Ω*). It is the set of sensor nodes chosen to compose the virtual sensors to respond the users’ queries.*

(5) $$\Omega =\{SN_{i,j}^{1},SN_{i,j}^{2},\cdots ,SN_{i,j}^{N_{\Omega }}\}$$

Thus, we formulate the provisioning task as an optimization problem, given by:

(6) $$\min\sum_{id=1}^{N_{Q}}\sum_{n=1}^{N_{\Omega }}\left(\kappa_{j,i,id}+\lambda_{j,i}+\chi_{j,i}\right)+\eta$$

Subject to:

1. *Sensors constraints*, which requires the selected sensors to satisfy all queries submitted by the middleware. This constraint can be stated as:
  (7) Φ(Ω)≥Φ(QUERY)
2. *Energy constraint*, which requires all sensors to have a minimum energy level (usually defined by the user). The Energy constraint can be stated as:
  (8) $$\lambda_{j,i}>minimum\_energy$$
  where j=1,⋯,|Query|; i=1,⋯,|Ω|; and minimum_energy>0.
3. *Coverage constraint*, which requires all selected sensors to be in the region of interest. This constraint is formulated as:
  (9) Cv(Ω,id)=True
  where
  Cv(⋯) returns True if the sensor nodes in Ω fully cover $R_{id}$, and False otherwise.
  and
  $$id=1,\cdots ,N_{N_{Q}}$$

## 4. The Proposed Approach

As cited earlier in Section 1, the provisioning task consists of selecting the physical sensor nodes to compose the user’s virtual sensors. However, as one of the main requirements, this process should avoid redundant nodes, in order to save WSN constrained resources. For that reason, ACxSIMv2 defines two phases to achieve a properly provisioning. In phase 1, an algorithm called *Adaptive Clustering Algorithm Based on Similarity* (ACASIMv2) performs clustering of the physical sensor nodes that are inside of the user’s interest region. The physical nodes are clustered based on the similarity of their measurements. Hence, all nodes within a specific cluster have measurements close to each other according to an acceptable error threshold parameter, defined by the user. This process is dynamic (adaptive) once the clusters can be recreated as the measurement values of nodes within a given cluster begin to exceed the acceptable error threshold. As the cluster formation is mainly controlled by the error threshold, there is no need to know in advance the number of clusters, as in k-means algorithm [46,47], for instance. In Phase 2, performed by the *Ant Colony Optimization for Sensor Selection Based on Similarity* (ACOSIMv2), only an optimal subset of physical nodes of each cluster is selected to form the virtual sensor requested by the user. This optimal subset attempts to minimize the energy consumption of physical sensors (Section 4.2). An ant colony optimization (ACO) system is used to perform this operation. The choice of an ACO system is made because of its successful application in several WSN previous works [27,48,49,50].

**Figure 4:**
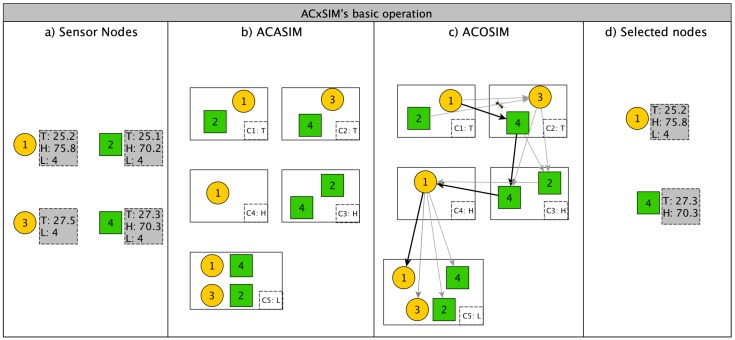
ACxSIMv2’s basic operation.

Figure 4a depicts an example of a sensor cloud comprised of two WSN from different providers ($WSN_{1}$ is represented by yellow circles and $WSN_{2}$ by green squares). The nodes are heterogeneous, which means that they do not have the same types of sensors. The middleware, based on the user’s request parameters, sends a query to the cloud. For example, consider that a user wants to receive the temperature, humidity, and light from a specific region which encompasses Nodes 1, 2, 3, and 4. Then, each node starts transmitting its measurements towards the middleware, using its sink node as a gateway. The middleware waits for a predefined length of time to receive the measurements from the nodes. A dashed grey square shows the sensors devices each node has and the values in given instant of time (T,H,L stands for Temperature, Humidity, and Light respectively). Then, it runs the ACASIMv2 algorithm, which is responsible for clustering the nodes. However, different from our previous work, ACASIMv2 clusters the nodes based on similarity of the nodes’ measurements for each dimension (variable). Hence, the same node will be in different clusters of different dimensions. For example, Figure 4b shows that Node 1 is in the same cluster as Node 2 in the temperature dimension, but alone in humidity dimension, while in super-group with all other three nodes in light dimension. After running ACASIMv2, the middleware records the formation of the clusters and starts ACOSIMv2 (Figure 4c). ACOSIMv2 defines a subset of nodes and which sensors devices will be used to measure the environment. (we will explore how ACOSIMv2 works in Section 4.2). These nodes will be active, while the others enter into a power-saving mode (in this work, the nodes in this mode will be considered as inactive). The active sensor nodes will send only the measurements of the sensors devices defined by ACOSIMv2. For example, Node 4 does not need to send its light measurements, since Node 1 represents it. This behavior will help to save energy from the nodes. The middleware will consider the measurements of the each active (selected) node to represent all others nodes inside of its clusters. This is possible since each cluster contains only nodes whose measurements are below a THRESHOLD defined by the user. In other words, the measurements of the inactive nodes can be predicted according to the active node of its cluster.

### 4.1. ACASIMv2—Adaptive Clustering Algorithm Based on Similarity

**Figure 5:**
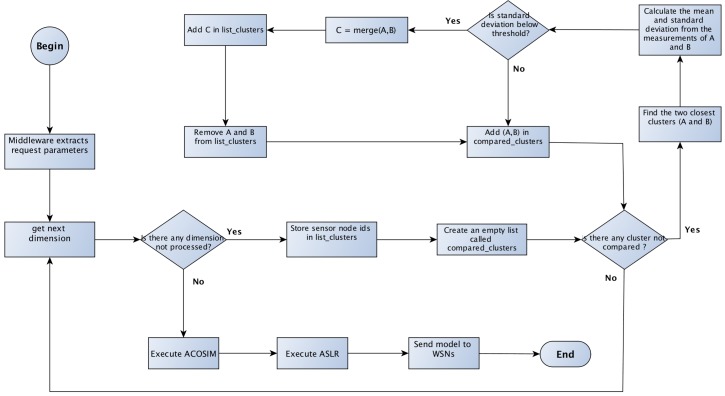
ACASIMv2 basic operation flowchart.

Figure 5 depicts how the ACASIMv2 works. First, the middleware receives a new user query $QUERY_{i}$. Then, the middleware searches in its local database for all active physical sensor nodes deployed in the interest region specified in the request parameters. Then, for each dimension, the middleware repeats the following procedure. Initially, each sensor node is treated as an individual cluster and its identifiers (ids) are added to a list called list_clusters. An empty list, called compared_clusters, is created and has the purpose of storing the clusters already compared. Next, the algorithm enters a loop and will be interrupted only when there are no longer any pairs of clusters that have not been compared. If there is at least one pair of non-compared clusters, the algorithm computes, based on Euclidean distance, the two nearest clusters (labeled *A* and *B*). Then, the algorithm takes the values of the measurements of the clusters’ nodes and computes the mean ($\bar{X}$) and the standard deviation (σ). If the value of σ is below a predefined THRESHOLD value for that dimension, then the two clusters are merged into another cluster labeled *C*. The new Cluster *C* is added to the list_clusters list and the set (A,B) is added to the compared_clusters list. This process is repeated until there are no more clusters to be compared. At the end of the clustering procedure, there will be clusters of sensor nodes for each dimensions, as depicted in Figure 4. In sequence, the middleware executes ACOSIMv2 to choose the sensor nodes to create the virtual sensors. The middleware then generates a model of the measurements, based on the ASLR algorithm [51], and sends to the sensors nodes. The objective is to exploits the temporal correlation between the measurements and executes ACASIMv2 as soon as there is some change in the environmental conditions. The sensor nodes, after received the models, compare the values of each new measurement with the value generated by the model. If there is a significant difference, the sensor node sends an alert to the middleware in order to rebuild the groups.

**Figure 6:**
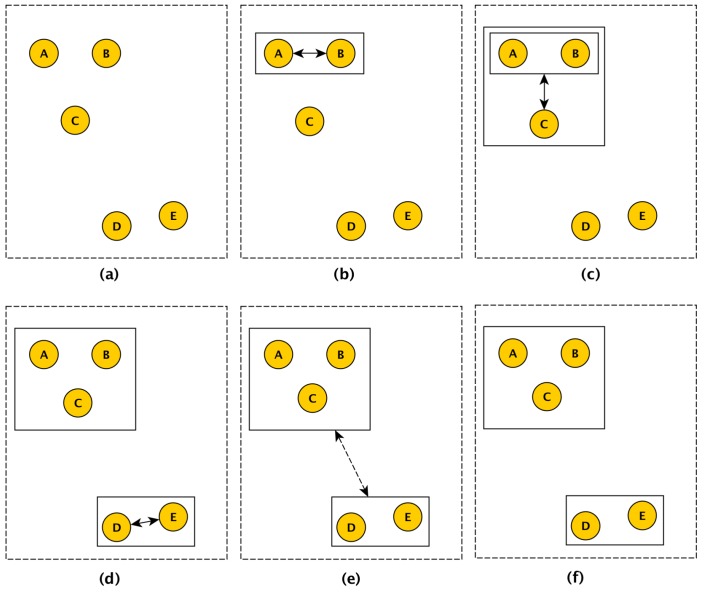
Basic operation of the ACASIMv2.

Figure 6 depicts how the clustering process works. For the sake of simplicity, it considers measurements from one dimension (i.e., temperature). There are five sensor nodes and the similarity of their measurements is represented by their relative position in the image (which does not necessarily correspond to their physical location). Thus, the closer the two sensor nodes, the more similar their measurements. Initially, the sensor nodes correspond to individual clusters (Figure 6a). Then, the ACASIMv2 estimates that *A* and *B* are the two closest groups and that the standard deviation of their measurement is below the predefined THRESHOLD for that dimension. In this case, *A* and *B* are merged into a new group (Figure 6b). In Figure 6c, the new group (A,B) is also merged with *C*, as the standard deviation of measurements *A*, *B*, and *C* was also below the threshold. As *D* and *E* are the two closest groups and the standard deviation of their measurements is also below the threshold, they are merged to form another group (Figure 6d). In Figure 6e, the ACASIMv2 attempts to merge the groups (*A,B,C*) and (*D,E*). However, as the standard deviation of measurements from all sensor nodes is above the threshold, the groups are not merged (Figure 6f).

Differently from our previous work [20], in ACxSIMv2, it is possible to configure different THRESHOLD values for each dimension, in order to reflect the difference between the range values and/or users’ interest. For example, considering the Green Orb dataset, where temperature ranges from 13 ${}^{\circ }C$ to 31 ${}^{\circ }C$ and humidity ranges from 18% to 55%, some user could accept an error of ±0.5 ${}^{\circ }C$ on temperature and ±2.0% on humidity.

### 4.2. ACOSIMv2—Ant Colony Optimization for Sensor Selection Based on Similarity

**Figure 7:**
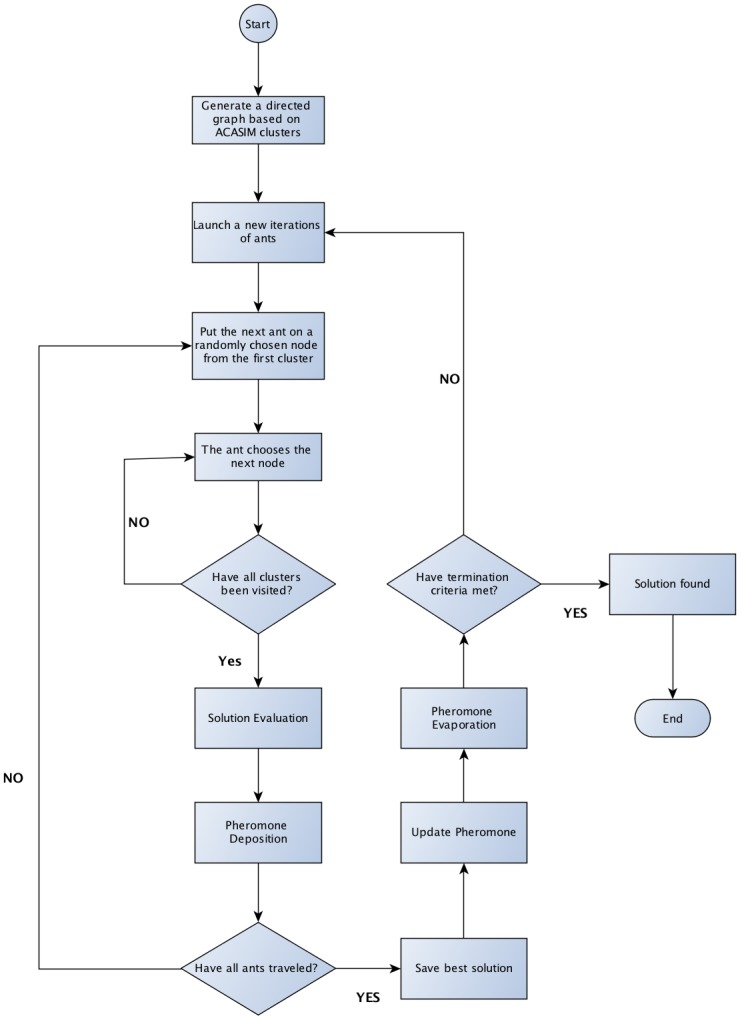
ACOSIM’s Flowchat.

This section explains how ACOSIMv2 finds the optimal set of sensor nodes which will form the user’s virtual sensors. The first subsection shows a brief review of Ant Colony Optimization (ACO). The latter subsections detail how ACOSIMv2 implements the two main steps of ACO system: (i) build the solution; and (ii) pheromone update. Figure 7 gives an overview of the whole process.

#### 4.2.1. Ant Colony Optimization

Ant colony optimization is a class of algorithms inspired by how some ant species forage for food [24]. The ants deposit a chemical substance, named pheromone, on the ground, which influences the choices they take. While spreading around the area, searching for food, the ants tend to use paths with higher pheromone concentration. Besides the complex behavior of foraging, other collective behaviors of real ants that have been proposed and applied include the division of labor, cemetery organization, brood care, and construction of nests. ACO has successfully been applied to several NP-hard optimization/combinatorial problems [52,53]. The emergence of shortest-path selection in foraging behavior is explained by the differential path length effect and autocatalysis (positive feedback, reinforcement learning through pheromone deposit, etc.) [54,55].

The ACO algorithm involves two basic procedures:

1. Procedure for building a solution. $N_{A}$ (the number of ants) ants build $N_{A}$ solutions to the problem.
2. Procedure for updating the pheromone concentration. The solutions are evaluated through an evaluation function in order to measure their quality. The update of the pheromone concentration is based on the evaluation function. Thus, better solutions cause more pheromone to be deposited in its region.

#### 4.2.2. Solution Construction Procedure

Usually, an ACO algorithm executes in terms of iterations. At the beginning of a new iteration, artificial ants are launched, and each of them is responsible for building a solution. A solution is defined by the path that a specific ant took from a source towards a final destination. At the end of the iteration, all solutions are evaluated, and the better solution is chosen. ACO repeats this process until found a termination criteria, also called stopping condition. The most common used termination criteria are: (i) maximum number of iterations is exceeded; (ii) an acceptable solution is found; and (iii) all ants start to follow the same path (stagnation behavior). The provisioning problem defines a solution as a subset of nodes taken from the set of all nodes in the sensor cloud. Since ACASIMv2 creates cluster of similar nodes, in order to build a solution, the ant just needs to pass through all clusters and select at least one node.

To explain how ACOSIMv2 runs, it will be used, as an example, a sensor cloud with five clusters created by ACASIMv2 (Figure 4b). Each cluster has a index *c* and also a sensor type *t* associated to it, since ACASIMv2 clusters sensor nodes by sensor types (or dimensions). Let $N_{C}$ be the number of clusters created by ACASIMv2 (In the given example, $N_{C}=5$) and $C_{c,t}$ ($1\leq c\leq N_{C}$ and t∈TYPE) be the set of sensor nodes ($SN_{i,j}$) inside the *c-th* cluster. In Figure 4b, $C_{1,T}=\left\{1,2\right\}$, $C_{2,T}=\left\{4,3\right\}$, $C_{3,H}=\left\{2\right\}$, $C_{4,H}=\left\{4,1\right\}$, and $C_{5,L}=\left\{1,2,3,4\right\}$, where *T*, *H*, and *L* stand for *Temperature*, *Humidity*, and *Light* respectively. Let G=(V,E) be a graph, with $V=\{SN_{i,j}|1\leq i\leq N,and\;1\leq j\leq N^{i}\}$, and *E* be the set of all the edges formed by the combination of nodes in $C_{x,y}$ and $C_{x+1,z}$, $1\leq x<N_{C}$, y∈TYPE, z∈TYPE (Figure 4c).

At each iteration, $N_{A}$ ants, starting from different nodes, begins its search process through the graph, as shown in Figure 4c (for the sake of simplicity, only one route taken by an ant is depicted). Let $\Omega_{it}^{a}\subset V$ be the solution found by the $a_{th}$ ant at the end of the $it_{th}$ iteration. At the beginning of the next iteration $\Omega_{it}^{a}=\left\{\emptyset \right\}$. Following the example, the ant starts its first interaction (a=1,it=1) at Node 1, from $C_{1,T}$, heading to Node 4 at Cluster $C_{2,T}$. After that, the ant starts exploring the next dimension, going to Node 4 at Cluster $C_{3,H}$, heading to 1, at $C_{4,H}$. Finally the ant gets to 1, at $C_{5,L}$, with $\Omega_{1}^{1}=\left\{1,4\right\}$. The middleware also associates which sensor types each node will deactivate. In the example, the sensor Node 4 does not need to monitor *Light* variable anymore, since its measurements can be represented by sensor Node 1. The choice of the edge is based on a function that calculates the probability of each edge (Equation (15)). Thus, edges with higher probability have greater chances of being selected. The input of this function is the amount of pheromone on the edge and a heuristic value that represents the knowledge related to the specific problem (see Section 4.2.4). The path is finalized when the ant has gone through all the clusters. At least one sensor node from each cluster will be part of the solution. Consequently, the virtual sensor will have a complete view of the region of interest. After all ants have performed their search, a procedure is executed in which the values of the pheromones of the edges are updated (Equation (12)). The amount of pheromone to be deposited is related to the cost of the path that passes through the edges. Thus, the better the path, the greater the amount of pheromone deposited at its edges. In the provisioning problem, the cost is given by Equation (6) (see Section 4.2.3. Furthermore, there is an evaporation process that decreases the amount of pheromone already deposited. After updating the pheromones on the edges, a new cycle is started; this continues until the termination criteria is reached. At the end of the last cycle, the best route (which is the most used) is considered as the solution (Ω) and nodes using this route are selected to compose the virtual sensor, as illustrated in Figure 4d.

#### 4.2.3. Pheromone Update Procedure

The ACO construction procedure leads ants to build their solutions by selecting vertices from the graph *G*. The ants select at least one physical sensor from all of the clusters formed. The success of the solution construction procedure relies on the design of pheromone deposition and heuristic information. The pheromone is deposited between every pair of physical sensors to record the historical desirability of assigning them to the solution to be constructed. In the provisioning problem, the Equation (6) represents this desirability, since we expect to minimise the equation as maximum as possible. This way, the pheromone deposited between an unassigned physical sensor (*f*) and an physical sensor node already in the solution (*e*) can be expressed as:

(10) $$\tau_{e,f}\left(t+n\right)=\rho \cdot \tau_{e,f}\left(t\right)+\Delta \tau_{e,f}$$

(11) $$\Delta \tau_{e,f}=\sum_{a=1}^{N_{A}}\Delta \tau_{e,f}^{a}$$

(12) $$\Delta \tau_{e,f}^{a}=\left\{\begin{array}{cc} Q/L_{a} & \mathrm{if}\;a_{\mathrm{th}}\;\mathrm{ant}\;\mathrm{used}\;\mathrm{the}\;\mathrm{edge}\;\left(e,f\right)\\ 0 & \mathrm{otherwise} \end{array}\right.$$

where:

- *e* is a sensor node already in the solution;
- *f* is an unassigned sensor node;
- $\tau_{e,f}\left(t\right)$ is the intensity of pheromone on the link (e,f) in time *t*;
- $\Delta \tau_{e,f}^{k}$ is the amount of pheromone deposited on the edge (e,f) by the $a_{th}$ ant within the time interval (t,t+n);
- *Q* is a constant;
- $L_{a}$ is value of Equation 6 considering the sensor nodes selected by the $a_{th}$ ant; and
- ρ is a constant smaller than 1; otherwise the pheromone would accumulate without bound (0.5 is recommended).

#### 4.2.4. Heuristic Information

The heuristic information is based on the number of selected nodes from each cluster. To ensure that the solution passes through all clusters, the nodes from the less used clusters have higher probability to be selected. Thus, the heuristic information is expressed by:

(13) $$\eta_{c}=\frac{1}{\varphi_{c}+0.1}$$

(14) $$\varphi_{c}=\frac{nOfN_{c}}{\sum_{x=1}^{N_{C}}nOfN_{x}}$$

where

- $N_{C}$ is the set of clusters created by ACASIMv2;
- $nOfN_{x}$ is the number of selected nodes from Cluster $x\in N_{C}$.

Once the pheromone level and heuristic information are defined, the probability of assigning an unassigned sensor *f* to the solution *S* is calculated by

(15) $$P_{e,f}\left(t\right)=\left\{\begin{array}{cc} \frac{{\left[\tau_{e,f}\left(t\right)\right]}^{\alpha }\cdot {\left[\eta_{e,f}\right]}^{\beta }}{\sum_{l}{\left[\tau_{e,f}\left(t\right)\right]}^{\alpha }\cdot {\left[\eta_{e,f}\right]}^{\beta }}, & \mathrm{if}\;f\;\in \;\{V-tabu_{a}\}\\ 0, & \mathrm{otherwise} \end{array}\right.$$

where

- *V* is the set of all vertices (nodes) in graph *G*;
- $tabu_{a}$ is a dynamically growing set of vertices (nodes) already visited by the $a_{th}$ ant.

## 5. Performance Evaluation

**Figure 8:**
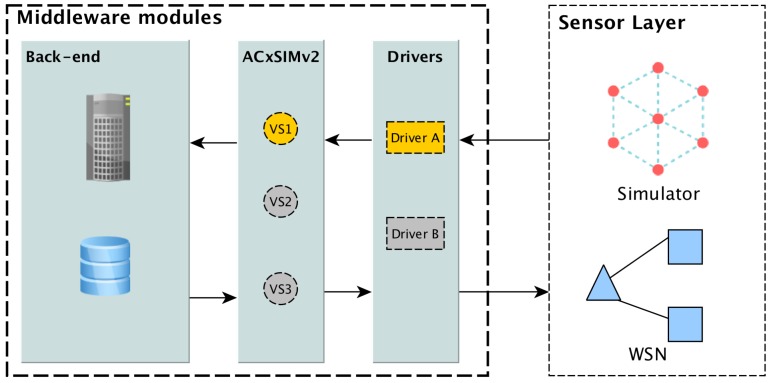
Middleware modularized architecture.

To evaluate ACxSIMv2, we have implemented a middleware, using Java language, according to the architecture described in Figure 8. The middleware is modularized to facilitate the sharing of its modules between different computers (servers). However, ours test have been performed on an Ubuntu Linux Operational System, running in VirtualBOX VM, with 8GB of memory RAM.

In Figure 8, the back-end module has two main components: a server and a database. The server receives the users queries and searches for virtual sensors already registered in the database. It is also responsible to instantiate ACxSIMv2 module when provisioning is necessary. ACxSIMv2 communicates with sensor layer via drivers located in the driver layers. For each WSN there must be a driver responsible to interface the WSN’s sink node. In this work, as we intend to evaluate ACxSIMv2 in scenarios with a large number of nodes, we choose to simulate WSN, using Sinalgo Framework (https://sourceforge.net/p/sinalgo/wiki/Home/). We have implemented a drive to interface Sinalgo simulations.

Although we have used a simulated WSN, the nodes generates real data obtained from two different datasets: (i) Intel Lab Data (http://db.csail.mit.edu/labdata/labdata.html); and (ii) Green Orbs Dataset [56,57]. Intel Lab dataset consists of the measurements of 54 sensor nodes measured every 31 s between 28 February and 4 April 2004. The Green Orbs Dataset contains the measurements from 271 nodes taken from sensor nodes deployed in a forest during 3–8 August 2011. For both datasets, we have considered the temperature, humidity, light, and battery voltage values. However, all nodes do not have the same number of measurements (possibly due to errors or failures in the sensor-reading process). Therefore, to properly evaluate the performance of the proposed solution, the same number of measurements is considered for each node. We have taken the first 5000 measurements for the Intel Lab dataset, and the first 248 for the Green Orbs. The original datasets contain missing or outliers/noisy values, which were interpolated with the average of the values from the previous and subsequent measurements. The summary of the deployments is shown in Table 2.

We have first evaluated ACxSIMv2 itself, focusing on how the performance metrics are affected by changes in the values of THRESHOLD parameter. Then, we have compared it with our previous work (ACxSIMv1) to verify how the new approach enhanced the clustering and sensor node selection process. Finally, we also have compared ACxSIMv2 with LEACH protocol [30]. In the LEACH protocol, only cluster heads communicate with the sink node, and each sensor node has probability *P* of being elected cluster head. The cluster heads receive all measurements from nodes in their clusters and generate an aggregate value from them. In this work, the *mean* is considered as the aggregation function. LEACH is considered because it is a classical protocol for cluster formation in traditional WSN. The single-hop (direct) communication [58] was used as a benchmark case. In the single-hop communication, each node transmits its measurements directly to the sink node. Considering the measurements transmitted by each node in the Intel Lab Dataset, the total energy consumption in the single-hop communication was 1122.92 mJ, and the total number of sent packets were 270,000. In the Green Orbs dataset the total energy consumption was 279.51 mJ, and the total number of sent packets were 67,208.

### 5.1. Performance Metrics

As explained earlier, ACxSIMv2 performs the provisioning task using two main algorithms. The first one, ACASIMv2, clusters the physical sensor nodes based on the similarity of their measurements and the later, ACOSIMv2, selects only a subset of nodes that minimize the energy consumption. Hence, the mean squared error (MSE) and energy consumption were chosen to evaluate ACxSIMv2 properly. As the created clusters may include sensor nodes not physically close to each other, MSE helps to investigate how reliable is the measurements sent by the selected sensors, i.e., how they can be representative of multiple regions. The energy consumption measures how well ACOSIMv2 selects the sensor nodes in order to save energy.

#### 5.1.1. Mean Squared Error (MSE)

Considering the measurement transmission of only a subset of nodes of each cluster represent all other nodes in the cluster, it is possible to calculate the mean squared error (MSE) of each cluster. That is, how much the other measurements (called $X_{o}$) differ from the measurements of the transmitting sensor nodes ($X_{T}$). In this way, it is possible to evaluate the accuracy of the measurements sent by each cluster. The overall accuracy of a specific set of clusters can be defined as the average of all clusters’ MSE and can be expressed as:

(16) $$MSE=1/N_{C}\sum_{c=1}^{N_{C}}1/|C_{c,t}|\sum_{o=1}^{|C_{c,t}|}\left|\right|X_{o}-X_{T}{\left|\right|}^{2}$$

#### 5.1.2. Energy Consumption

For the purpose of consumption estimation, the energy model addressed in [51,59] is used. The transmission rate of 0.26 μ bit/s is considered, the electric current flowing through the node to receive a package is considered as 7.0 mA while that needed to transmit is considered as 21.5 mA. Thus, the following model is defined:

- $Q_{Transmission}=3\;*\;21.5\;mA\;*\;(0.26\;*\;10^{-6}\;bit/s\;*\;Data\_Length$)
- $Q_{Receive}=3*7.0\;mA*\left(0.26*10^{-6}\;bit/s*Data\_Length\right)$
- $Q_{Listen}=3\;*\;7.0\;mA*\left(0.26\;*\;10^{-6}\;bit/s\;*\;104\;bits\right)=0.00056784\;mJ/message$

where

- DissipatedEnergy(Q)=Voltage∗ElectricCurrent∗Time
- Time=TransmissionRate∗Data_Length.

**Figure 9:**
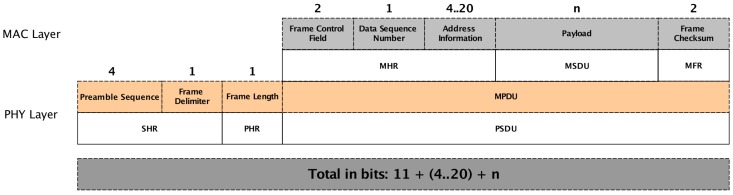
802.15.4 Data Frame.

We have considered a data frame format as defined in 802.15.4 MAC Layer [60] (Figure 9). We also have considered that a sensor node in listening mode only needs to receive the data frame header (104 bits). The energy spent in transmission or receiving mode is dependent on the data frame length. Since ACxSIMv2 choose which sensor device each sensor node will use, the length of the packets may be different. In our scenarios, all physical sensors nodes monitor four different variables (temperature, humidity, light and its energy level). Therefore, an active sensor node could transmit a data frame up to four variables of 4 bytes, yielding data frames with 152, 184, 216, or 248 bits. The sensors nodes start with energy level of 2 J.

### 5.2. Performance of ACxSIMv2

ACxSIMv2 was simulated in eight different scenarios varying the THRESHOLD parameter values. As cited in Section 4.1, ACxSIMv2 uses THRESHOLD to control cluster formation based on the standard deviation of the nodes’ measurements. In our previous work [20], we have used the following values: 0.1, 0.3, 0.5, 1.0, 2.0, 3.0, 4.0, and 5.0. However, since in ACxSIMv2 accepts THRESHOLD values for each dimension, we considered the values summarized in Table 3:

Values close to 0 means no clustering, because there is no acceptable difference between measurements, and high values of THRESHOLD could lead to groups with high variance and, subsequently, the creation of virtual sensors not representative of the physical sensor nodes. Therefore, the measurements from these virtual sensors could not meet the users’ interest.

**Figure 10:**
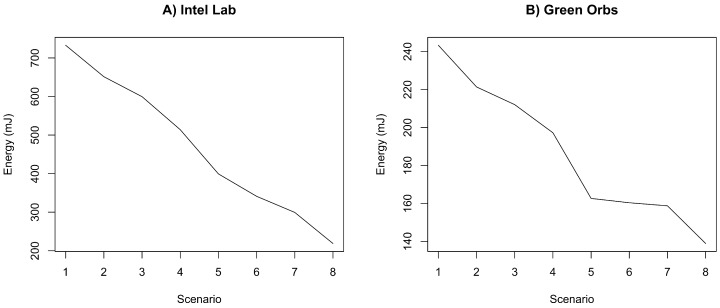
Energy consumption of ACxSIMv2 in the simulated scenarios for Intel Lab and Green Orbs Dataset.

Figure 10 shows the energy consumption in mJ for each scenarios in both datasets. It is clear that there is a negative correlation between the THRESHOLD and Energy consumption variables, i.e., as the THRESHOLD value increases, the total energy consumption decreases. This is due to the fact that high values of THRESHOLD generate fewer clusters with more nodes in each cluster. Moreover, since ACxSIMv2 selects only a subset of sensor nodes from each cluster, there is a tendency of less nodes’ activities (the total number of packets decreases). This behavior influences the overall energy consumption of the sensor cloud, since transmission is the activity the most draw current from the batteries in a sensor node [59].

**Figure 11:**
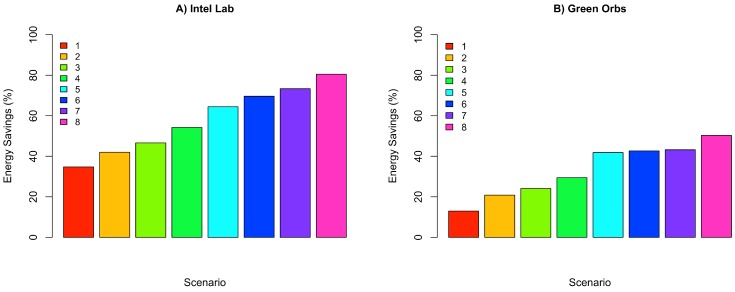
Percentage of energy consumption savings of ACxSIM for Intel Lab and Green Orbs Dataset.

**Figure 12:**
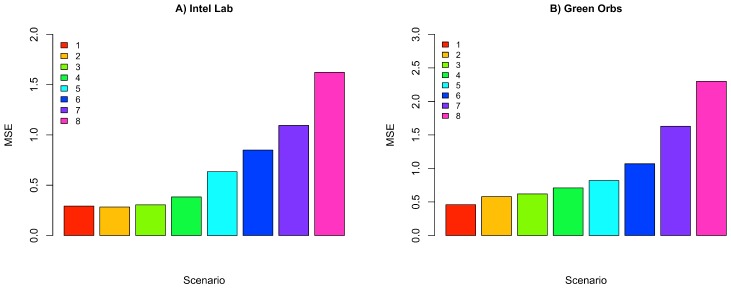
MSE of ACxSIMv2 for Intel Lab and Green Orbs Dataset.

**Figure 13:**
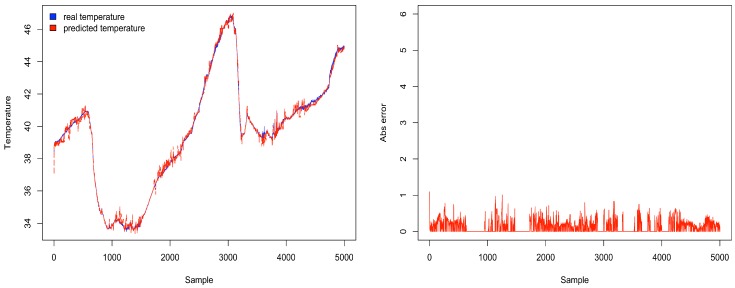
Real and predicted temperatures (${\;}^{\circ }C$) of Node 18 with THRESHOLD equal to 0.5.

**Figure 14:**
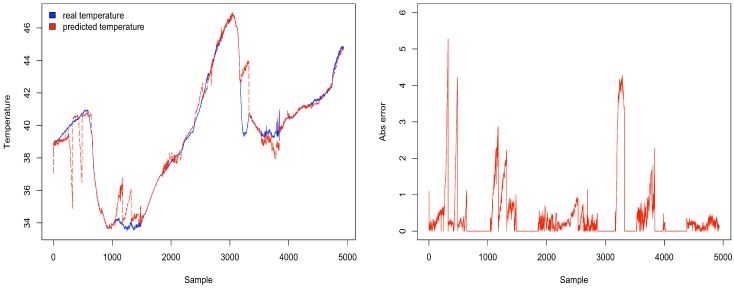
Real and predicted temperatures (${\;}^{\circ }C$) of Node 18 with THRESHOLD equal to 5.0.

Figure 11 depicts the percentage energy consumption savings with ACxSIMv2 compared to single-hop communication. As THRESHOLD value increases, the number of physical nodes per group also increases, causing a decrease in the number of groups. Since only a subset of nodes in each group responds, there is a decrease in the number of packet transmissions in the network, causing a reduction in energy consumption. However, although higher values of THRESHOLD seem to be the best configuration, because of the crescent energy savings, the MSE should be considered to properly select the THRESHOLD value. Since this parameter influences the similarity between the nodes’ measurements in a cluster, the error (deviation) between real and transmitted data is also affected. Therefore, MSE is used to track how reliable are the measurements received by the middleware. To obtain the MSE, we have considered the the errors from the measurements of all dimensions. Figure 12 shows an increasing tendency in MSE as the value of THRESHOLD rises. As THRESHOLD represents the acceptable standard deviation in the cluster nodes’ measurements, high values of THRESHOLD increase the error (deviation) in the MSE metric. Figure 13 and Figure 14 reproduce the error between the real measurements from Intel Lab Node 18 and the measurements associated with its clusters (predicted data) during the simulations in the scenarios with THRESHOLD equals to 0.5 and 5. While in a cluster, Node 18 may be selected to transmit its measurements or could be put in a lower energy consumption state. Figure 13 points out that the predicted data closely follows the real sensed data. Most of the time, the error is within the limit of $0.5{\;}^{\circ }C$ (with some occasional values up to 1.0). Similar results have also been observed for all the remaining nodes. However, in Figure 14, it is clear that the absolute error falls beyond $2{\;}^{\circ }C$ in most of the time (with some occasional values up to 5). Therefore, this configuration may not be suitable for real WSN applications.

### 5.3. Comparing ACxSIMv2 and ACxSIMv1

Regarding ACxSIMv1, we have used the results from [20]. Each scenario uses the same THRESHOLD parameter for all the dimensions, since, in ACxSIMv1, it is not possible to have different values. The simulations considered only the temperature and humidity from the Intel Lab dataset. Therefore, we have simulated ACxSIMv2 again using the same set of configurations. Table 4 summarizes the results from ACxSIMv1 and ACxSIMv2.

From the results, we observe that ACxSIMv2 overcome ACxSIMv1 in all scenarios, which was expected since ACxSIMv2 enhanced the clustering and selection procedure. Regarding the energy consumption, as ACxSIMv2 chooses not only the physical sensor nodes but also the sensor devices it will use, the length the transmitted packets will be proportional the measurements taken, which explains why the overall energy consumption of the cloud is reduced.

### 5.4. Comparing ACxSIMv2 and LEACH

The LEACH protocol was simulated in eight different scenarios considering only the Intel Lab Dataset. In each scenario, the value of *P* was modified. As cited earlier, *P* defines the probability of a node being elected as the cluster head. The values of *P* used were 0.2, 0.3, 0.4, 0.5, 0.6, 0.7, 0.8, and 0.9. Table 5 summarizes the results from the simulation.

**Figure 15:**
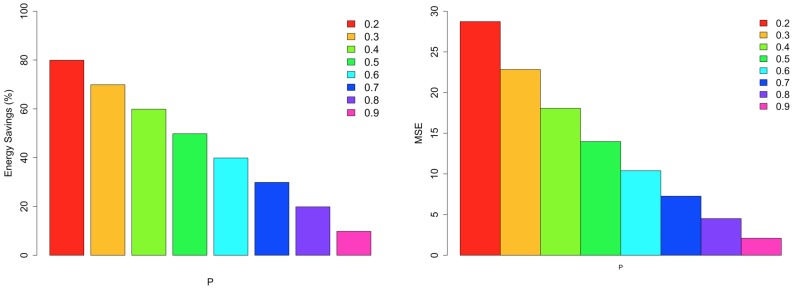
Energy savings and MSE of LEACH for Intel Lab Dataset.

Figure 15 shows the percentage energy consumption savings and the MSE of LEACH in the eight simulated scenarios. Considering the figure, MSE is closely correlated with the value of *P*, which was expected since, in the LEACH protocol, the clusters are created according to the nodes’ proximity (based on physical distance). Small values of *P* means that few cluster heads are selected. In this case, the MSE is higher since each cluster may contain nodes distant from each other, resulting in clusters with measurements that are very different. As the value of *P* increases, the number of cluster heads increases as well. Hence, the MSE decreases because it contains more nodes close together (based on physical distance). Regarding the energy consumption savings, as the value of *P* increases, more nodes are elected as cluster heads, which increases the number of transmissions inside the network. Consequently, the energy consumption savings decrease. As the value of *P* approaches 1, the energy consumption of LEACH becomes more similar to that of the single-hop communication. It is clear from the aforementioned figures that higher values of *P* are not necessary the best configurations, since the energy consumptions tend to be higher.

**Figure 16:**
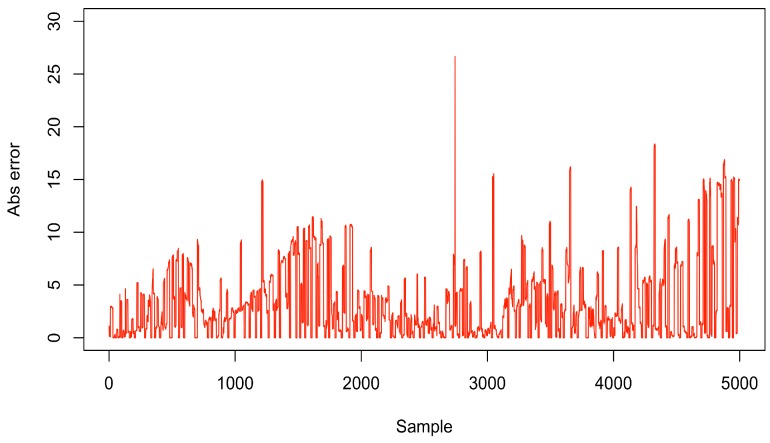
Absolute error in LEACH scenario with P equals to 0.2.

According to Table 4 and Table 5, the highest value of MSE in ACxSIMv2 (1.05) is about 27× lower than highest value of MSE in LEACH scenarios (28.72). In this same configuration, the energy consumption savings of ACxSIMv2 (69.50%) is just approximately 1.15× lower than LEACH. It is important to stress that this is just the scenarios with highest energy savings in both solutions. However, a MSE equals to 28.72 makes LEACH, with P=0.2, infeasible for WSN applications. As depicted in Figure 16, the absolute error between the real data from Node 1 and the data associated with its clusters, during the simulations in the scenarios with P=0.2, falls beyond $5{\;}^{\circ }C$ in most of the time. Considering the results with the lowest MSE, LEACH has a MSE equals to 2.09 and energy savings equals to 9.80% for P=0.9, while ACxSIMv2 has 1.05 and 69.50% for the same metrics in the scenario with THRESHOLD=6.0. Therefore, ACxSIMv2 has better performance in both MSE and energy consumption metrics, about 2.0× and 7×, respectively.

## 6. Discussion

We briefly present some trade-off of our proposed approach. Since we focused on sensed data and the prediction of the data using correlations and regression, ACxSIMv2 is recommended only for monitoring type application, i.e., all sensor nodes periodically gather information by monitoring phenomena on a specific geographic area. Other types of applications would require adjusts mainly in the system model. In *Target-detection* (or Classification) applications [46], for instance, the signal power would be an interesting decision variable to incorporate to the model.

ACxSIMv2 tries to capture the dynamics of data correlation by considering a regression predictor on each sensor node. As soon as one sensor node detects that the error between the sensed data and the predicted one is beyond a threshold, it warns the middleware to reconstruct the clusters again. However, in a highly dynamic environment, regarding changes in phenomena conditions or the position of the physical nodes (mobile nodes), it would be required the execution of ACxSIMv2 consecutive times in a short period of time. In this case, the energy consumption of the proposed approach would not be efficient, since each sensor node could predict only a few measurements between the executions of ACxSIMv2.

We also have not considered priority of applications/queries. To provide priority, i.e., applications that must be execute before orders, the system model, given by Equation 6 should be adapted.

In our problem definition (Section 3), we have defined 2D spatial in accordance with the dataset used in our simulations. We have chosen to use Intel Lab and Green orbs dataset since they are popular dataset in WSN literature. Both dataset provides only the x and y coordinates of the sensor nodes (in meters relative to the upper right of the map); however, it is important to emphasize that the whole approach is not dependent on the dimensionality of the spatial cells adopted.

## 7. Conclusions

Provisioning of virtual sensors is one of the foremost tasks in sensor cloud environments. It is defined by the selection and allocation of physical sensors nodes to respond queries from different users. Traditionally, most middlewares for sensor cloud proposed in the literature consider the selection of all physical sensor in the region of interest, leading to wasting of energy consumption. Recent works tried to explore correlation and prediction in homogenous environments which is not appropriate for heterogeneity environment of sensor clouds. In this paper, we presented ACxSIMv2, an extension of our previous work, named ACxSIMv1, to enhance provisioning of virtual sensors performed by middlewares in sensor cloud environments. ACxSIMv2 selects a optimal subset of heterogeneous sensor nodes, based on users’ request, to create the required virtual sensors.

ACxSIMv2 comprises two algorithms: ACASIMv2 and ACOSIMv2. The first, ACASIMv2, clusters heterogeneous sensor nodes based on the similarity of their measurements. It differs from traditional approaches in literature since most works cluster homogeneous sensor nodes usually based on the physical distance between them. As ACASIMv2 use data (measurements) similarity, the clusters may be compounded by nodes from different areas. However, ACASIMv2 guarantees that all nodes have measurements below some threshold. In that sense, a small subset from each cluster could represent multiples areas. The second algorithm, ACOSIMv2, takes place after the clusters formation. Based on an Ant Colony Optimization algorithm, ACOSIMv2 selects a subset of sensor nodes from each created cluster. The selection procedure is performed under three main guidelines: (i) all hetegeneous nodes together are able to process user’s requests, (ii) minimize the number of selected nodes, and (iii) minimize the overall energy consumption the sensor cloud.

We have performed simulations to evaluate the performance of ACxSIMv2. First, we have compared it with single-hop communication. Although a simplistic solution, single-hop is the standard approach used by classical works in sensor clouds literature. Then, we have compared ACxSIMv2 with LEACH protocol, a classical solution for cluster formation in traditional WSN. Finally, we also have compared it with our previous work (ACxSIMv1).

Simulations have shown that ACxSIMv2 has better performance than the LEACH protocol regarding energy consumption (about 7.0×) and MSE (2.0×), indicating the feasibility of the proposed algorithm. These results are explained by the fact that ACxSIMv2 can significantly reduce the amount of traffic in the network (by selecting sensor nodes whose measurements represents multiple geographical areas) while maintaining the measurements error below some desired threshold. In addition, the simulations have shown that ACxSIMv2 has been able to attend the applications requirements in two different environments. The first one (Intel Lab Dataset) considered a small WSN deployed in a indoor office (a controllable environment). The second scenario (Green Orbs Dataset) represents a large WSN deploy in a forest (harsh environment). Our approach will be useful when a large number of heterogeneous sensors nodes are deployed in near future with the popularization of sensor cloud paradigm. By reducing the associated energy consumption of those nodes, the WSN will operate much longer without interruptions.

We intend to enhance ACxSIMv2 by: (i) performing simulations in more complex environments, taking into account other variables such as the routing protocol; (ii) comparing ACxSIMv2 with others clustering algorithms; (iii) implementing the algorithm on a hardware platform to assess its performance in a real environment; and (iv) integrating ACxSIMv2 with the interactive model proposed in [40,41,42].

## Figures and Tables

**Table 1:** Notations used in the work.

Notation	Description
**A. Sets and Variables**	
SC	Sensor cloud: a set of Wireless Sensor Networks (WSN)
$WSN_{i}$	Set of sensor nodes (SN) of the $i_{th}$ WSN
$SN_{i,j}$	The $j_{th}$ sensor node of the $i_{th}$ WSN. A SN comprises a set of sensor (*S*)
$S_{k}^{i,j}$	The $k_{th}$ sensor of $SN_{i,j}$. It defines a set of $M^{k,i,j}$ measurements gathered by the sensor
$C_{c,t}$	Set of sensor nodes ($SN_{i,j}$) inside the *c-th* cluster of type *t*
Ω	Set of selected sensor nodes (SN) to be provisioned
$\Omega_{it}^{a}$	Solution found by the $a_{th}$ ant at the end of the $it_{th}$ iteration
$type_{i}$	a specific type/dimension that represents a monitored physical condition
TYPE	The set of all dimensions monitored by SC
QUERY	The set of all queries triggered by sensor cloud’s users.
$T_{i}$	Deployed area of $WSN_{i}$, denoted by $H_{i}\times W_{i}$. *H* and *W* means height and width
*R*	Region of interest
**B. Dimensions**	
*N*	Number of WSN in the SC
$N^{i}$	Number of SN in the $WSN_{i}$
$D^{i,j}$	Number of sensors devices of the $SN_{i,j}$
$N_{T}$	Number of all types of physical variables the SC can monitor
$M^{k,i,j}$	Number of measurements of the $k_{th}$ sensor device of $SN_{i,j}$
$N_{Q}$	Number of triggered queries
$N_{C}$	Number of clusters created by ACASIMv2
$|C_{c,t}|$	Number of sensor nodes inside the c−th cluster
$N_{\Omega }$	Number of selected sensor nodes
$N_{A}$	Number of Ants
**C. Functions**	
Φ	Function to compute the number of unique sensor types present in a set
Cv	Function to test if the sensors nodes in Ω fully cover *R*

**Table 2:** Deployment summary.

Deployment	Nodes	# Data	Sensing Interval	Type
Intel Lab	54	5000	31 segs.	indoor
Green Orbs	271	248	10 min.	outdoor

**Table 3:** Simulated scenarios.

Scenario	Temperature	Humidity	Light
1	0.1	1.0	100
2	0.3	1.5	150
3	0.5	2.0	200
4	1.0	2.5	250
5	2.0	3.0	300
6	3.0	3.5	350
7	4.0	4.0	400
8	5.0	5.0	500

**Table 4:** ACxSIMv1 and ACxSIMv2 results.

Threshold		Energy (mJ)			Energy Savings (%)			MSE	
		v1	v2		v1	v2		v1	v2
0.1		1289.77	753.39		1.09	32.90		1.01	0.29
0.3		1256.28	688.15		3.66	38.71		0.96	0.29
0.5		1155.60	654.14		11.38	41.74		0.97	0.28
1.0		903.87	582.21		30.68	48.15		1.46	0.31
2.0		702.25	493.04		46.14	56.09		1.83	0.38
3.0		605.91	433.53		53.53	61.39		2.07	0.48
4.0		554.62	397.78		57.46	64.57		2.10	0.60
5.0		306.30	342.40		66.52	76.84		2.50	1.05

**Table 5:** LEACH protocol results.

P	Energy (mJ)	Energy Savings(%)	MSE
0.2	26,227.10	79.91	28.72
0.3	39,334.82	69.88	22.83
0.4	52,422.89	59.86	18.06
0.5	65,567.48	49.80	13.98
0.6	78,544.14	39.86	10.39
0.7	91,599.68	29.86	7.26
0.8	104,681.71	19.85	4.50
0.9	117,804.67	9.80	2.09
